# Identification of hepatocellular carcinoma-related subtypes and development of a prognostic model: a study based on ferritinophagy-related genes

**DOI:** 10.1007/s12672-023-00756-6

**Published:** 2023-08-09

**Authors:** Ganggang Wang, Jian Li, Lingkang Zhu, Zhijie Zhou, Zenghui Ma, Hao Zhang, Yulong Yang, Qiang Niu, Xiaoliang Wang

**Affiliations:** 1 Department of Hepatobiliary Surgery, Shanghai Pudong Hospital, Fudan University Pudong Medical Center, Shanghai, 201399 China; 2 Endoscopy Center, Minhang Hospital, Fudan University, Shanghai, 201199 China; 3 Jing’an District Central Hospital, Fudan University, Shanghai, 200040 China; 4 Institute of Gallstone Disease, Center of Gallbladder Disease, Shanghai East Hospital, Tongji University School of Medicine, Shanghai, 200120 China; 5 Department of General Surgery, Shidong Hospital, Shidong Hospital Affiliated to University of Shanghai for Science and Technology, Yangpu District, Shanghai, 200438 China; 6 Department of General Surgery, Qingpu Branch of Zhongshan Hospital, Fudan University, Shanghai, China

**Keywords:** Ferritinophagy, Single-cell RNA sequencing (scRNA-seq), Liver hepatocellular carcinoma (LIHC), Immune, Prognosis, TCGA

## Abstract

**Background:**

Hepatocellular carcinoma still has a high incidence and mortality rate worldwide, and further research is needed to investigate its occurrence and development mechanisms in depth in order to identify new therapeutic targets. Ferritinophagy is a type of autophagy and a key factor in ferroptosis that could influence tumor onset and progression. Although, the potential role of ferritinophagy-related genes (FRGs) in liver hepatocellular carcinoma (LIHC) is unknown.

**Methods:**

Single-cell RNA sequencing (scRNA-seq) data of LIHC were obtained from the Gene Expression Omnibus (GEO) dataset. In addition, transcriptome and clinical follow-up outcome data of individuals with LIHC were extracted from the The Cancer Genome Atlas (TCGA) dataset. FRGs were collected through the GeneCards database. Differential cell subpopulations were distinguished, and differentially expressed FRGs (DEFRGs) were obtained. Differential expression of FRGs and prognosis were observed according to the TCGA database. An FRG-related risk model was constructed to predict patient prognosis by absolute shrinkage and selection operator (LASSO) and COX regression analyses, and its prognosis predictive power was validated. Ultimately, the association between risk score and tumor microenvironment (TME), immune cell infiltration, immune checkpoints, drug sensitivity, and tumor mutation burden (TMB) was analyzed. We also used quantitative reverse transcription polymerase chain reaction (qRT-PCR) to validate the expression of key genes in normal liver cells and liver cancer cells.

**Results:**

We ultimately identified 8 cell types, and 7 differentially expressed FRGs genes (ZFP36, NCOA4, FTH1, FTL, TNF, PCBP1, CYB561A3) were found among immune cells, and we found that Monocytes and Macrophages were closely related to FRGs genes. Subsequently, COX regression analysis showed that patients with high expression of FTH1, FTL, and PCBP1 had significantly worse prognosis than those with low expression, and our survival prediction model, constructed based on age, stage, and risk score, showed better prognostic prediction ability. Our risk model based on 3 FRGs genes ultimately revealed significant differences between high-risk and low-risk groups in terms of immune infiltration and immune checkpoint correlation, drug sensitivity, and somatic mutation risk. Finally, we validated the key prognostic genes FTH1, FTL, using qRT-PCR, and found that the expression of FTH1 and FTL was significantly higher in various liver cancer cells than in normal liver cells. At the same time, immunohistochemistry showed that the expression of FTH1, FTL in tumor tissues was significantly higher than that in para-tumor tissues.

**Conclusion:**

This study identifies a considerable impact of FRGs on immunity and prognosis in individuals with LIHC. The collective findings of this research provide new ideas for personalized treatment of LIHC and a more targeted therapy approach for individuals with LIHC to improve their prognosis.

## Introduction

Liver hepatocellular carcinoma (LIHC) is among the most widely occurring tumors around the globe, with high morbidity and mortality rates. According to the latest statistics [1], LIHC is the fourth most prevalent cancer, and in China, the disease leads to the second highest mortality rate. More than 1 million individuals are expected to die from LIHC in 2030, and the 5-year survival rate of LIHC is 18%, which is the second most fatal tumor after pancreatic cancer [2]. The main treatment modality for LIHC is currently surgical resection. However, most patients are not suited for resection surgery due to limitations such as the size and location of the tumor and liver dysfunction [3]. For patients with advanced, unresectable LIHC, targeted therapy combined with monotherapy is the first-line treatment option [4]. Nevertheless, LIHC still has a high mortality rate. Therefore, a deeper understanding of disease biology is necessary to find novel therapeutic approaches [5].

Ferritinophagy is a type of autophagy and an essential factor in ferroptosis. As a cellular self-degradation mechanism, autophagy is a conserved catabolic cellular process. Autophagy helps in the lysosomal degradation of cellular proteins and damaged organelles, which in turn helps in the recycling and protection to ensure the maintenance of cellular homeostasis and stress response. Autophagy is categorized into three main types: microautophagy, macroautophagy, and chaperone-mediated autophagy [6]. Ferroptosis is a type of programmed cell death that is highly associated with lipid metabolism and reactive oxygen species (ROS) [7]. The process is closely associated with autophagy and cancer. As a new ferroptosis-related autophagic process, ferritinophagy is an intracellular process and mechanism linking ferroptosis and cancer. Moreover, ferritinophagy could influence tumorigenesis and progression [8–11].

Single-cell RNA sequencing (scRNA-seq) data of LIHC were retrieved from the GEO database. In addition, transcriptome and clinical follow-up outcome data of individuals with LIHC were extracted from the TCGA dataset. Differential cell subpopulations were distinguished and differentially expressed ferritinophagy-related genes (DEFRGs) among immune cells were identified. Subsequently, cell types with high scores were selected for functional enrichment analysis of their differentially expressed genes (DEGs). The differential expression of FRGs and their prognosis were analyzed using the TCGA dataset. Subsequently, the association between immune cell infiltration and DEFRGs was investigated. A nomogram and calibration curves were drawn on the basis of the DEFRGs as well as other pathological features. In addition, the model results were visualized. Finally, drug sensitivity analysis and TMB analysis were performed. This study helps better understand the pathogenesis of LIHC and promotes the development of targeted therapeutic strategies for patients with LIHC to improve their prognosis.

## Materials and methods

### Data acquisition

From the GEO official website (https://www.ncbi.nlm.nih.gov/geo/) [12], the single-cell transcriptome (single-cell sequencing, scRNA-seq) dataset GSE149614 [13] was extracted. The species was *Homo sapiens*, and GPL24676 Illumina NovaSeq 6000 was utilized for detection. Transcriptome data from 10 of these LIHC tissues and 8 normal liver tissue samples were extracted and included in this study. The relevant clinical information of the patients is presented in Table 1.

**Table 1:** Clinical characteristics of patients in the GSE149614 and TCGA-LIHC

Characteristic	GSE149614	TCGA-LIHC
Sex, n (%)		
Female		120 (32.60)
Male		248 (67.39)
Age, median (IQR)		61 (51–69)
Family history of cancer, n (%)		
Yes		110 (29.89)
No		207 (56.25)
Unknown		51 (13.85)
AJCC stage, n (%)		
Stage I	3 (30.00)	171 (46.46)
Stage II	1 (10.00)	85 (23.09)
Stage III/IV	6 (60.00)	88 (23.91)
Unknown		24 (6.52)
Survival status, n (%)		
Living		129 (35.05)
Dead		239 (64.94)

Transcriptome and somatic mutation data were extracted from The Cancer Genome Atlas (TCGA) database of the LIHC project, which contains transcriptome (mRNA) data from 368 LIHC tumor tissues and 50 paraneoplastic control tissues, and somatic mutation data from 366 tumors. All above sample data and the corresponding clinical features and follow-up outcome data of all patients were included in this study.

### Quality control of single-cell data

The expression matrix of the GSE149614 dataset was created as a Seurat object utilizing the R package Seurat 4.2.0 [14]. The proportion of mitochondrial genes in all genetic material indicates the homeostatic state of the cell. In general, a higher percentage of mitochondrial genes compared to all other genes might put the cell in a stressful state. Therefore, cells with > 20% mitochondrial gene content were filtered. Since low-quality cells or empty droplets have fewer genes, double cells exhibit an unusually high number of genes. Therefore, cells with FEATURES < 500 or > 6000 were also filtered. Ultimately, transcriptome data for 58,127 cells were obtained.

The sequencing depth of the GSE149614 dataset was normalized by the "NormalizeData" function with the default "LogNormalize" method. The 2000 variable features of the dataset were detected by the "FindVariableFeatures" function using the "vst" method. Subsequently, the data were scaled utilizing the "ScaleData" to exclude the effect of sequencing depth. Principal Component Analysis (PCA) was utilized to find the significant principal components (PC), and the P-value distribution was observed using the Elbowplot function. Finally, 22 PCs were screened out by t-Distributed Stochastic Neighbor Embedding (tSNE) analysis for dimensionality reduction. The K-nearest neighbors of Euclidean distance in the base PCA space are constructed using the default parameters of "FindNeighbors" and the 22 PC dimension parameters. The "FindClusters" function and the "cluster" function were utilized to obtain the optimal resolution. Finally, the cells were categorized into 21 clusters with a resolution of 0.5. Finally, the "RunTSNE" function is used for dimensionality reduction to allow visualization and exploration of the dataset.

### Cell type annotations

The single-cell data GSE149614 were subjected to cell type annotation using the SingleR dataset from the R package SingleR 2.0.0 [15], yielding eight cell types: T cells, B cells, endothelial cells, monocytes, smooth muscle cells, dendritic cells, macrophages, and hepatocytes. Subsequently, the classification results were verified using marker genes of these 8 cell types. The expression of marker genes in various cells was displayed using the "DotPlot" and "FeaturePlot" functions. The marker genes for T cells, endothelial cells, monocytes, macrophages, B cells, smooth muscle cells, dendritic cells, and hepatocytes were CD3D, CD79A, PECAM1, CD14, CD68, ACTA2, FLT3, ALC, and ALC, respectively. The "FindAllMarkers" function was used to identify DEGs between cell types. This function implements the Wilcoxon rank-sum test and compares the gene expression of one cell type with the gene expression of all other cells.

### FRG expression in immune cell subpopulations

The GeneCards database (https://www.genecards.org/) [16] delivers extensive information about human genes. Therefore, it was used to collect FRGs. In total, 21 FRGs were determined using "ferritinophagy" as a search terminology. Subsequently, 20 FRG s were retained by excluding the missed genes during the probe transformation of the dataset, including USP24, PCBP1, ATG16L1, ATG7, SNCA, FBXW7, TRIM7, TNF, ATG5, WDR45, CYB561A3, FTH1, NCOA4, HERC2, ALOX15. BECN1, ELAVL1, ZFP36, BCAT2, and FTL.

The FRGs were intersected with the DEGs across immune cells (T cells, B cells, dendritic cells, monocytes, and macrophages) to obtain DEFRGs across immune cells. The expression of these DEFRGs in various cells is presented by the "DoHeatmap" function. The Pearson correlation coefficient of DEFRGs was calculated using the "rcorr" function. Subsequently, the correlation heat map was plotted using the "corrplot" function. The expression profile of DEFRGs in different cell types was displayed using the "FeaturePlot" function.

### FRG scoring and enrichment analysis

The expression of DEFRGs was scored for each cell of the single-cell dataset GSE149614 using the AUCell package [17]. Subsequently, the cell types with higher scores (AUCs) were selected for functional enrichment analysis of their DEGs.

The Kyoto Encyclopedia of Genes and Genomes (KEGG) [18] is a commonly used dataset for storing data about genomes, biological pathways, diseases, and drugs. Gene ontology (GO) [19] functional annotation analysis is an extensively used method of performing large-scale functional enrichment studies of genes involving biological process (BP), molecular function (MF), and cellular component (CC). GO and pathway KEGG enrichment analyses were conducted using the clusterProfiler package [20] for DEGs in a subpopulation of cells with high FRG scores in the single-cell dataset GSE149614. In addition, adj. P-value < 0.05 indicated statistical differences.

### Cellular communication analysis

Intercellular communication was inferred and quantified using the CellChat package [21] by combining single-cell expression profiles with known receptors, ligands, and their cofactors. Significantly interacting ligand-receptor relationship pairs were found by ligand–receptor interaction probability and perturbation tests. The cell–cell communication network was then integrated by summing the number or strength of ligand–receptor relationship pairs that significantly interact between cell types. The number and intensity of interactions were demonstrated using heat maps and circle plots, respectively. All significant receptor–ligand pairs during immune cell signaling were counted using bubble plots.

### Differential expression and correlation analysis of FRGs in TCGA-LIHC data set

Differential expression of FRGs was examined for TCGA-LIHC transcriptome data using Wilcoxon and presented in box plots. Heat maps of DEFRGs were drawn utilizing the ggplot2 package [22] and the pheatmap package [23] to visualize the expression of FRGs in the samples. Subsequently, the correlation between DEFRGs was analyzed using Pearson correlation, where significantly correlated ones were shown with dotted line plots.

### Immune cell infiltration analysis

CIBERSORT could deconvolute the transcriptome expression matrix as per the principle of linear support vector regression to determine the abundance and composition of immune cells in a mixture of cells. The counts of TCGA-LIHC dataset gene expression matrix was uploaded to the CIBERSORTx (https://cibersortx.stanford.edu/) [24] online analysis tool to calculate by utilizing the LM22 feature gene matrix, we calculated the immune cell infiltration status of the samples. The analysis was conducted using the tool's default parameters with a permutation of 1000. Subsequently, the obtained results were filtered to include only samples with a significance level (p-value) below 0.05. As a result, we obtained the matrix data for immune cell infiltration.the immune cell infiltration of samples according to the LM22 signature gene matrix to filter the output p < 0.05 samples, yielding immune cell infiltration matrix data. Subsequently, the Wilcoxon test was utilized to compare the difference in the extent of immune cell infiltration between tumor and paracancerous tissues. Pearson correlation analysis of the infiltration levels between various immune cells was performed. Differential immune cells were observed between the tumor and paraneoplastic tissue groups. Therefore, the correlation between these cells and the DEFRGs of the single-cell dataset GSE149614 was analyzed and visualized using a heat map.

### Weighted gene co-expression network analysis (WGCNA)

DEGs in tumor and paracancerous tissues in the TCGA-LIHC dataset were identified as per a linear model utilizing the limma package in R language [25], with DEG screening criteria of adj. p value < 0.05 and |log2FC|> 1. Weighted Gene Co-Expression Network Analysis (WGCNA), a systems biology method to characterize gene association patterns between various samples, could detect highly synergistic gene sets and candidate biomarkers or therapeutic targets according to the endogeneity of the gene set and the connection between the phenotype and the gene set. WGCNA was carried out using the WGCNA package [26] on DEGs obtained from the analysis of DEGs associated with the TCGA-LIHC dataset. Initially, the correlation coefficient between any two genes was measured, and the linkage between genes in the network was made to obey a scale-free network using the weighted values of the correlation coefficients. Afterward, a hierarchical clustering tree was created based on the correlation coefficients between genes. Various clustering tree branches correspond to different gene modules (different colors represent different modules), followed by the calculation of module significance. The minimum number of module genes was set to 25, softpower was set to the optimal soft threshold of 4, and the module merge shear height was set to 0.25. Ultimately, the link between the extracted modules and differentially infiltrated immune cells and DEFRGs was analyzed.

### Prognostic marker screening

Based on the screened DEFRGs, COX regression analysis was utilized to assess the correlation between gene expression and prognostic survival of individuals with LIHC in the TCGA-LIHC dataset. The Least absolute shrinkage and selection operator (LASSO) technique, a compressed estimation [27], obtains a developed model by creating a penalty function, which could compress some coefficients while setting some coefficients to zero. Hence, it retains the benefit of subset shrinkage and is a biased estimator that deals with data having complex covariance. Prognostic markers were screened using LASSO regression. Subsequently, the variables were screened utilizing the Glmnet function of the glmnet package [28] and cross-validated using the cv.glmnet function. Finally, the combination of prognostic markers that minimized the CV coefficient was screened.

### Risk score construction and assessment of clinical prognosis predictive power

The risk score for every patient in the TCGA-LIHC dataset was measured with the stated equation:

$$\mathrm{risk score}={\sum }_{i=1}^{n}{\mathrm{Coefficient}}_{i}\times {mRNA \,Expression(gene}_{i})$$

The Coefficient is the LASSO regression coefficient, and mRNA expression is the expression level of the gene (log2 conversion).

The maxstat package [29] was utilized to measure the best cutoff value (cutoff) for the predictive ability of the risk score on survival time in individuals with LIHC. Subsequently, the diseased individuals were categorized into high-/low-risk groups based on this cutoff value. Survival curves were plotted by means of the Kaplan–Meier (K–M) method. Risk scores were utilized to predict patients' 1-, 3- and 5-year survival with the aid of the SURVIVALROC package [30]. Ultimately, the predicted ROC was plotted, and AUC values were calculated.

The effect of other clinical features on patient prognosis and survival, including age, gender, family history of tumor, and tumor stage, was assessed using the Cox proportional risk model. Subsequently, forest plots were drawn using the forestmodel package [31]. Clinical features with a considerable effect on prognosis were included in multivariate Cox regression as covariates to assess if risk scores could independently predict patient prognosis, followed by forest plotting. The fit effect of the different models was assessed with AUC values.

A nomogram and calibration curves for the optimal multivariate model were plotted utilizing the rms package [32]. The model outcomes were visualized to empower predictive model results with higher readability. Ultimately, a consistency index (C-index) was measured to evaluate the power of the nomogram in predicting patient survival.

### Immunological analysis of the prognostic model

The abundance of immune cells between the high- and low-risk groups were compared by the Wilcoxon test based on the outcomes of immune infiltration of LIHC samples from the TCGA-LIHC database. Additionally, variations in the expression of common immune checkpoints (BTLA, CD40, CD70, CTLA4, HAVCR2, IDO1, LAG3, LMTK3, PDCD1, TIGIT, TJP1, TNFRSF14, TNFRSF18, and TNFRSF9) between both risk groups of the TCGA-LIHC dataset were evaluated by the Wilcoxon test.

### Drug sensitivity prediction

Drug response prediction was carried out by means of the R package oncoPredict 0.2 [33]. According to the Cancer Genome Project (CGP) database, the half-maximum inhibitory concentration (IC50) was determined for each patient using Ridge regression. The accuracy of the prediction was calculated by tenfold cross-validation. A linear model was utilized for comparing the variations in drug sensitivity between both risk groups utilizing the limma package in R [25]. The drug screening criteria for differential sensitivity were adj. p value < 0.05 and |log_2_FC|> 0.5.

### Somatic mutation analysis

The "mafCompare" function in the R package Maftools 2.14.0 [34] was utilized to perform Fisher's exact test for all genes in both risk groups of the TCGA-LIHC dataset to identify differentially mutated genes. Subsequently, oncoplot waterfall plots of FRGs were plotted. The tumor mutational load was compared by the Wilcoxon test between both risk groups and between the mutation and non-mutation FRG groups, and the results were presented as violin plots.

The sigminer package [35] was utilized to assess the mutation features of LIHC patient tumor somatic cells. The optimal number of mutation features was automatically extracted using the "sig_auto_extract" function, yielding eight mutation features. Subsequently, the extracted mutation features were matched with those in the Catalogue of Somatic Mutations in Cancer (COSMIC) database. The comparison of the variations in the expression of each mutation feature between both risk groups was carried out by the Wilcoxon test, and the results were presented in box plots.

### Cell culture, RNA extraction, reverse transcription, and quantitative PCR (RT-qPCR)

All cells were obtained from the Cell Bank of the Chinese Academy of Sciences. LO2 cells represent normal human liver cells, while HepG2, 97-H, and LM3 cells are immortalized liver cancer cells derived from patients. All cell lines were cultured in DMEM supplemented with 10% fetal bovine serum (Invitrogen, Carlsbad, CA, USA) at 37 ℃ in a 5% CO_2_ atmosphere.

Quantitative reverse transcription polymerase chain reaction (qRT-PCR) was performed to test the transcript abundances of FTH1、FTL. TRIzol (Invitrogen, Shanghai, China) reagent was employed for isolation of total RNA from the LO2, HepG2, 97-H, and LM3 cells. Using PrimeScript^™^ RT Master Mix (Perfect Real Time) (Takara Bio), the extracted RNA was reverse transcribed. Subsequently, Real-Time PCR was performed using TB Green Premix Ex TaqII (Tli RNaseH Plus) (Code No. RR820A) (Takara Bio) with the same conditions as specified in the kit. ABI 7900HT Real-Time PCR system (Applied Biosystems Life Technologies, CA, USA) were performed in triplicate. The data was analyzed by the 2^−△△^CT method. The primers used to test the expression of selected FTH1、FTL:

FTH1-F Sequence (5′to 3′): CCCCCATTTGTGTGACTTCAT;

FTH1-R Sequence (5′to 3′): GCCCGAGGCTTAGCTTTCATT;

FTL-F Sequence (5′to 3′): CAGCCTGGTCAATTTGTACCT;

FTL-R Sequence (5′to 3′): GCCAATTCGCGGAAGAAGTG;

### Hematoxylin and eosin (HE) staining, as well as the detection of FTH1 and FTL protein expression in cancer tissue wax blocks, using immunohistochemistry

First, sequentially place the paraffin sections in environmentally friendly dewaxing solutions I and II for 20 min each. Then, immerse them in absolute ethanol I and II for 5 min each, followed by 75% ethanol for 5 min. Rinse with water. Next, immerse the sections in hematoxylin staining solution for 3–5 min, followed by rinsing with tap water. Differentiate using a differentiation solution, rinse with tap water, counterstain with a bluing reagent, and rinse under running water. Then, sequentially immerse the sections in 85% and 95% gradient ethanol for 5 min each for dehydration, followed by staining with eosin solution for 5 min. Finally, sequentially place the sections in absolute ethanol I, II, and III for 5 min each, followed by clearing with xylene I and II for 5 min each. Mount using a neutral mounting medium. Finally, examine the sections under a microscope and capture images for analysis.

Cancer tissue wax blocks from 60 HCC patients were deparaffinized and subjected to antigen retrieval. The tissue chip is from the Servicebio biological sample library and was conducted under the approval of the Ethics Committee of our Hospital. Endogenous peroxidase was blocked using hydrogen peroxide solution and serum was added for blocking. The primary antibodies used include: Rabbit anti-FTH1 (dilution 1:100, #DF6278, Affinity) and Rabbit anti-FTL (dilution 1:100, #DF6604, Affinity) antibodies. The secondary antibody used was HRP-conjugated Goat anti-Rabbit (dilution 1:200, #GB23303, Servicebio). DAB was used for color development, followed by dehydration and counterstaining with hematoxylin. The Servicebio imaging analysis system was used to read the tissue measurement area automatically and calculate the H-score (which converts the number and staining intensity of positive cells in each slide into corresponding numerical values, with larger values indicating stronger comprehensive positive intensity).

### Statistical analysis

All data calculations and statistical analyses were carried out utilizing R 4.1.0 (https://www.r-projectt.org/). Multiple testing adjustment was conducted by means of Benjamini-Hochberg (BH). False discovery rate (FDR) adjustment was performed in multiple testing to reduce the false positive rate. Comparisons between two groups of continuous variables were performed with independent Student t-tests to estimate the statistical significance of normally distributed variables. Variations between non-normally distributed variables were assessed by the Mann–Whitney U test (i.e., Wilcoxon rank sum test). The predictive power of prognostic markers was assessed using Cox regression models. Receiver operating characteristic (ROC) curves were plotted utilizing the pROC package of R. In addition, the area under the ROC curve (AUC) was a measure to determine the risk score’s accuracy in predicting prognosis. All statistical p-values were two-sided tests, with P < 0.05 taken as statistically significant.

## Results

### Cellular heterogeneity of LIHC tissues

**Fig. 1:**
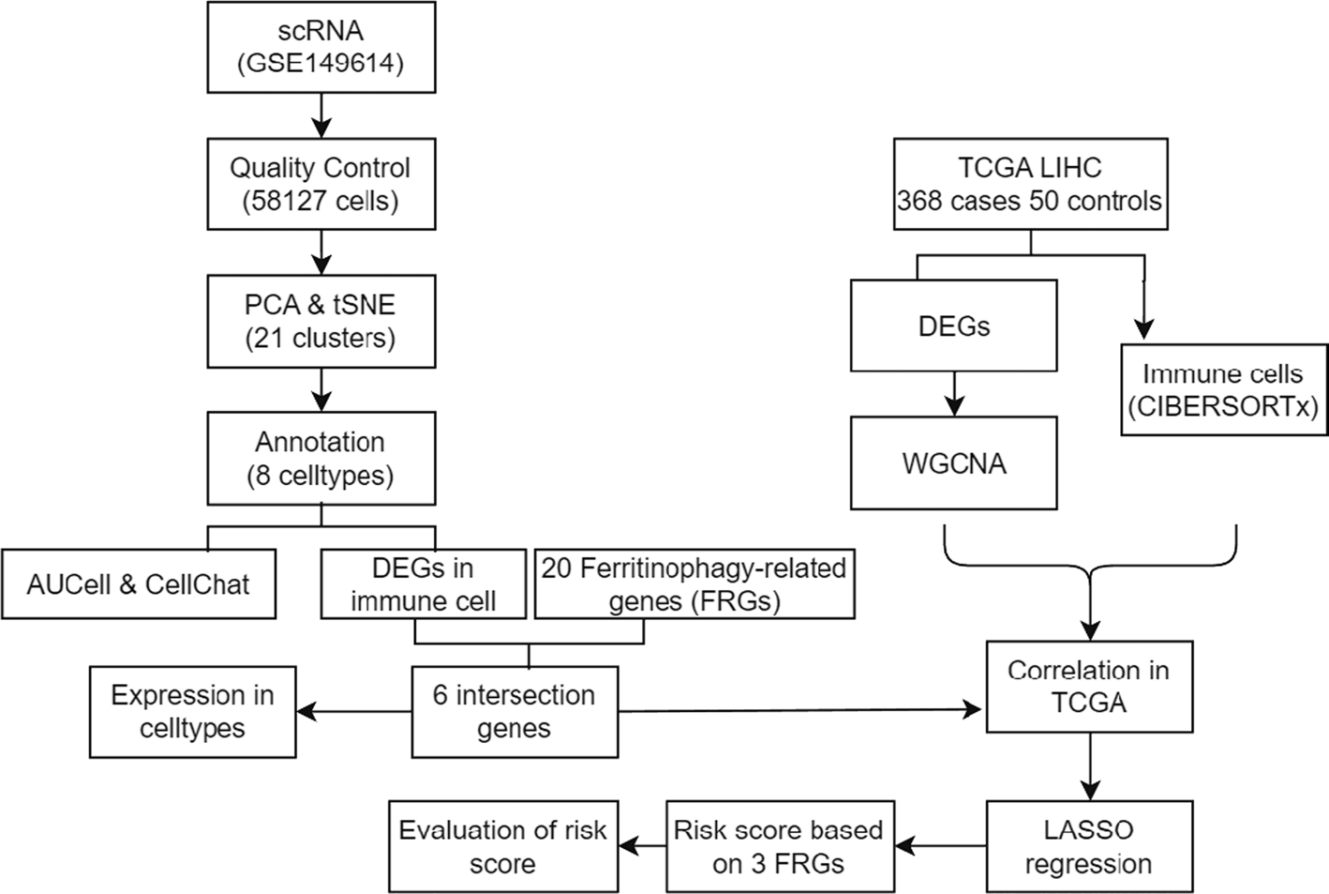
The study flow chart. *scRNA* Single-cell RNA-sequencing, *PCA* Principal Component Analysis, *tSNE* t-Distributed Stochastic Neighbor Embedding, *FRGs* FRGs, *TCGA* The Cancer Genome Atlas, *LIHC* Liver Hepatocellular Carcinoma Collection, *DEGs* Differentially Expressed Genes, *WGCNA* Weighted Gene Co-Expression Network Analysis, *LASSO* Absolute Shrinkage and Selection Operator

**Fig. 2:**
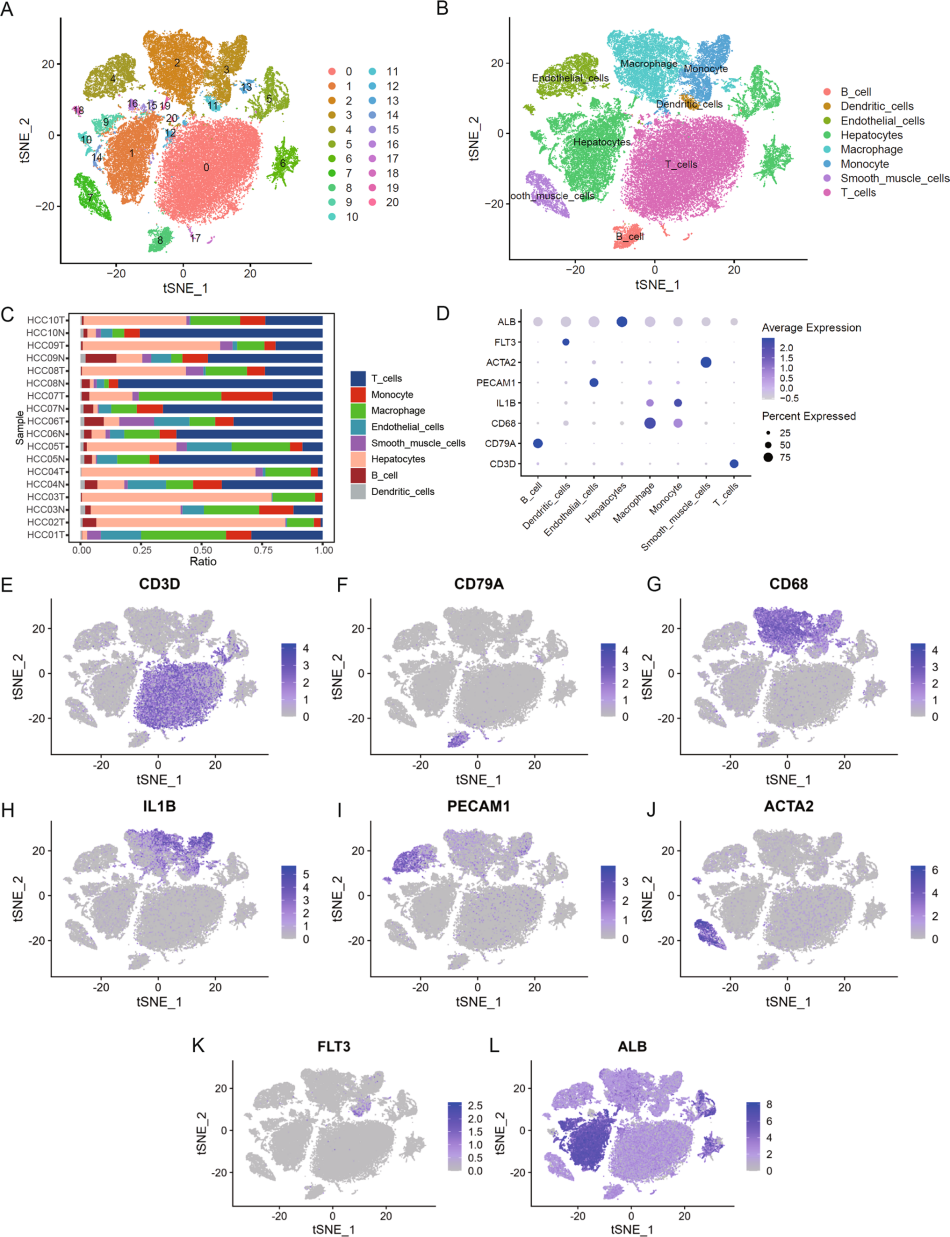
Identification of tissue cell subpopulations in LIHC patients on the basis of single-cell RNA-seq data set GSE149614. **A** A total of 57,836 cells were clustered into 19 cell clusters by tSNE. **B** Cells were annotated by singleR into 8 cell types: B cells, T cells, endothelial cells, monocytes, macrophages, smooth muscle cells, dendritic cells, and hepatocytes. **C** Percentage stack graph showing the proportion of 8 cell types in each sample. **D** Expression levels of marker genes for the 8 cells are shown as bubble plots, with darker colors indicating higher expression levels and larger circles indicating a higher percentage of gene expression within the cell population. **E**–**L** tSNE plots showing the expression levels of CD3D (**E**), CD79A (**F**), CD68 (**G**), CD14 (**H**), PECAM1 (**I**), ACTA1 (**J**), FLT3 (**K**), and ALB (**L**) in the single-cell data set

The flow chart of this study was shown in Fig. 1. Quality control was carried out on the GSE149614 dataset. In total, 57,836 cells were obtained after filtering cells with > 20% mitochondrial gene content, features < 500, or features > 6000 and visualized by tSNE downscaling. The 57,836 cells were successfully classified into 21 independent clusters (Fig. 2A). SingleR was utilized to identify the cell clusters in a total of 8 cell types (Fig. 2B). Among them, clusters 0, 12, and 20 were annotated as T cells (21,443, 37.07%); clusters 8 and 17 were annotated as B cells (1675, 2.89%); cluster 11 was annotated as dendritic cells (564, 0.97%); clusters 4 and 18 were annotated as endothelial cells (3788, 6.54%); clusters 1, 5, 6, 9, 10, 14, 15, 16, 19 were annotated as hepatocytes (15,979, 27.62%); cluster 2 and13 were annotated as macrophages (8506, 14.70%). Cluster 3 was annotated as monocytes (4021, 6.95%); cluster 7 was annotated as smooth muscle cells (1860, 3.21%). The proportion of each cell between each sample is shown in Fig. 2C. The marker genes of eight cell types were used (T cells: CD3D; B cells: CD79A; Endothelial cells: PECAM1; Monocytes: IL1B; Macrophages: CD68; Smooth muscle cells: ACTA2; Dendritic cells: FLT3; Hepatocytes: ALB) were plotted in a bubble map (Fig. 2D). Each marker gene had a high expression and cellular expression ratio in cell subpopulations, indicating the good auto-annotation effect of SingleR. tSNE plots showed CD3D (Fig. 2E), CD79A (Fig. 2F), CD68 (Fig. 2G), CD14 (Fig. 2H), PECAM1 (Fig. 2I), ACTA2 (Fig. 2J), FLT3 (Fig. 2K), and ALB (Fig. 2L) expression at the cellular level.

### Differential expression and scoring of FRGs in immune cells

**Fig. 3:**
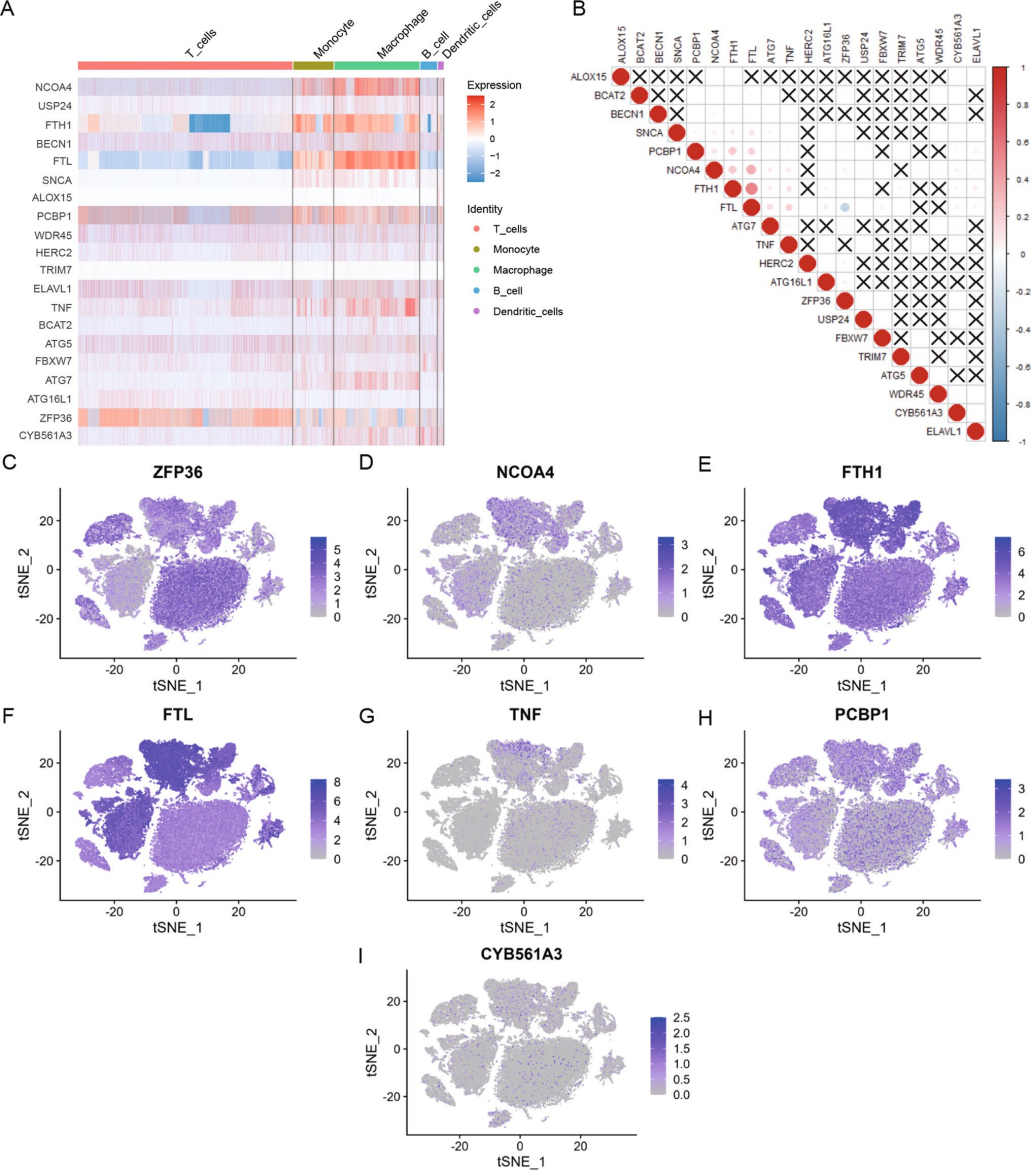
Expression levels and correlation analysis of FRGs among immune cells in the single-cell dataset GSE149614. **A** Heat map of the expression of 20 FRGs among immune cells. The top annotation bar indicates five immune cell types: T cells, macrophages, dendritic cells, B cells, and monocytes. Light-to-dark color gradient represents the progressively elevated expression level, with red color suggesting a positive relationship and blue color suggesting a negative relationship. **B** Heat map of the correlation of 20 FRGs, where red color denotes a positive relationship and blue color denotes a negative relationship. **C**–**I** tSNE plots showing the expression levels of ZFP36 (**C**), NCOA4 (**D**), FTH1 (**E**), FTL (**F**), TNF (**G**), PCBP1 (**H**), and CYB561A3 (**I**) in the single cell data set

The DEGs among immune cells from GSE149614 data were intersected with 20 FRGs, yielding 7 DEFRGs (ZFP36, NCOA4, FTH1, FTL, TNF, PCBP1, CYB561A3). The heat map was utilized to show the expression of 20 FRGs in immune cells (T cells, B cells, monocytes, macrophages, and dendritic cells) (Fig. 3A). ZFP36 was highly expressed in T cells, FTH1, FTL, PCBP1, NCOA4 in monocytes, FTL, FTH1, NCOA4, and TNF overexpressed in macrophages, and CYB561A3 was highly expressed in B cells. In addition, correlation analysis was performed for 20 FRGs. A considerably positive link was observed between FTH1 and FTL, and a considerably negative link between FTL and ZFP36 (Fig. 3B). Subsequently, seven DEFRGs were analyzed, including ZFP36 (Fig. 3C), NCOA4 (Fig. 3D), FTH1 (Fig. 3E), FTL (Fig. 3F), TNF (Fig. 3G), PCBP1 (Fig. 3H), and CYB561A3 (Fig. 3I) in the single-cell dataset. In addition to the highly expressed cell types shown in the heat map, ZFP36, FTH1, FTL, and PCBP1 were also widely expressed in other cell types, with FTH1 and FTL both having high expression in hepatocytes.

### Scoring and functional enrichment analysis of FRGs

**Fig. 4:**
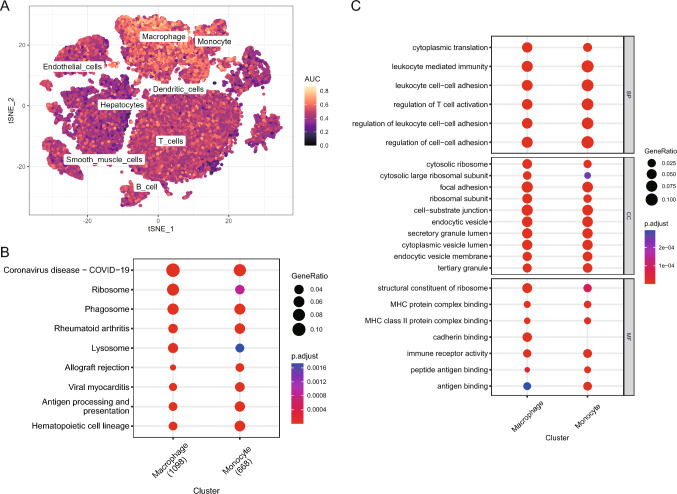
Single-cell data set GSE149614 FRG cell scoring and functional enrichment analysis of high-scoring cell populations. **A** FRG scores among cell subpopulations (AUC), with lighter colors indicating higher scores, where monocytes and macrophages had the highest mean scores. **B** Bubble plot of KEGG results for DEGs between monocytes and macrophages, with closer colors to red indicating smaller p and larger bubbles indicating more DEGs enriched within that pathway. **C** BP, CC, and MF enrichment results of GO analysis of DEGs between monocytes and macrophages presented as bubble plots, with colors closer to red indicating smaller p and larger bubbles indicating more DEGs enriched within that pathway. AUC area under the curve, *KEGG* Kyoto Encyclopedia of Genes and Genomes, *DEGs* differentially expressed genes, *GO* Gene Ontology, *BP* biological process, *CC* cellular component, *MF* molecular function

The expression of DEFRGs was scored for each cell in the single-cell dataset GSE149614 using the AUCell package (Fig. 4A). Monocytes and macrophages had the highest gene scores for FRGs with mean scores of 0.52 and 0.55, respectively. Subsequently, DEGs between these two cell types were subjected to functional enrichment analysis.

According to KEGG analysis results, DEGs of macrophages were primarily enriched in Coronavirus disease, ribosomes, phagosomes, rheumatoid arthritis, and lysosome pathways. DEGs of monocytes were primarily enriched in hematopoietic cell lineage, viral myocarditis, antigen processing and presentation, rheumatoid arthritis, and allograft rejection pathways (Fig. 4B). According to GO analysis results, DEGs of macrophages were primarily correlated with biological processes (BPs) such as cytoplasmic translation, leukocyte mediated immunity, and leukocyte cell–cell adhesion, cell components (CCs) such as cytosolic ribosome, cytosolic large ribosomal subunit, focal adhesion, and molecular functions (MFs) such as structural constitution of ribozyme, MHC protein complex binding, and MHC class II protein complex binding. DEGs of monocytes were primarily correlated with BPs, such as regulating the activation of T cells, cell–cell adhesion, and leukocyte mediated immunity, CCs such as endocytic vesicle membrane, endocytial vesicles, and tertiary granules, and MFs such as MHC protein complex binding, immune receptor activity, and MHC class II protein complex binding (Fig. 4C). Tables 2, 3 display the specific KEGG and GO enrichment results for Macrophage and Monocyte.

**Table 2:** Results of KEGG analysis based on differentially expressed genes of Macrophage and Monocyte

Cluster	ID	Description	GeneRatio	BgRatio	p.adjust	Count
Macrophage	hsa05171	Coronavirus disease—COVID-19	122/1098	232/8192	4.62E−45	122
Macrophage	hsa03010	Ribosome	88/1098	158/8192	9.03E−35	88
Macrophage	hsa04145	Phagosome	71/1098	152/8192	8.13E−22	71
Macrophage	hsa05323	Rheumatoid arthritis	46/1098	93/8192	3.50E−15	46
Macrophage	hsa04142	Lysosome	53/1098	132/8192	7.97E−13	53
Macrophage	hsa05140	Leishmaniasis	37/1098	77/8192	9.17E−12	37
Macrophage	hsa05330	Allograft rejection	24/1098	38/8192	5.41E−11	24
Macrophage	hsa05416	Viral myocarditis	31/1098	60/8192	5.41E−11	31
Macrophage	hsa04612	Antigen processing and presentation	36/1098	78/8192	5.41E−11	36
Macrophage	hsa05134	Legionellosis	30/1098	57/8192	5.41E−11	30
Monocyte	hsa04640	Hematopoietic cell lineage	38/668	99/8192	1.49E−14	38
Monocyte	hsa05416	Viral myocarditis	29/668	60/8192	1.85E−14	29
Monocyte	hsa04612	Antigen processing and presentation	33/668	78/8192	1.85E−14	33
Monocyte	hsa05323	Rheumatoid arthritis	36/668	93/8192	1.85E−14	36
Monocyte	hsa05330	Allograft rejection	22/668	38/8192	3.26E−13	22
Monocyte	hsa05332	Graft-versus-host disease	23/668	42/8192	3.41E−13	23
Monocyte	hsa04940	Type I diabetes mellitus	23/668	43/8192	5.81E−13	23
Monocyte	hsa05417	Lipid and atherosclerosis	54/668	215/8192	8.59E−13	54
Monocyte	hsa04659	Th17 cell differentiation	36/668	108/8192	1.91E−12	36
Monocyte	hsa05166	Human T-cell leukemia virus 1 infection	54/668	222/8192	2.74E−12	54

**Table 3:** Results of GO analysis based on differentially expressed genes of Macrophage and Monocyte

Cluster	ONTOLOGY	ID	Description	GeneRatio	BgRatio	p. adjust	Count
Macrophage	BP	GO:0002181	Cytoplasmic translation	102/1810	161/18903	1.12E−59	22
Macrophage	BP	GO:0002443	Leukocyte mediated immunity	131/1810	463/18903	9.92E−28	23
Macrophage	BP	GO:0007159	Leukocyte cell–cell adhesion	121/1810	414/18903	4.99E−27	21
Macrophage	BP	GO:0050863	Regulation of T cell activation	112/1810	376/18903	9.31E−26	20
Macrophage	BP	GO:1903037	Regulation of leukocyte cell–cell adhesion	112/1810	377/18903	9.64E−26	20
Macrophage	BP	GO:0022407	Regulation of cell–cell adhesion	131/1810	490/18903	1.51E−25	21
Macrophage	BP	GO:0002683	Negative regulation of immune system process	122/1810	449/18903	2.09E−24	15
Macrophage	BP	GO:0002253	Activation of immune response	111/1810	397/18903	3.23E−23	15
Macrophage	BP	GO:0002697	Regulation of immune effector process	107/1810	379/18903	9.70E−23	17
Macrophage	BP	GO:0002449	Lymphocyte mediated immunity	104/1810	367/18903	3.07E−22	15
Macrophage	CC	GO:0022626	Cytosolic ribosome	84/1848	105/19869	2.54E−64	21
Macrophage	CC	GO:0022625	Cytosolic large ribosomal subunit	50/1848	59/19869	2.73E−40	13
Macrophage	CC	GO:0005925	Focal adhesion	138/1848	422/19869	1.70E−39	18
Macrophage	CC	GO:0044391	Ribosomal subunit	88/1848	188/19869	6.66E−39	8
Macrophage	CC	GO:0030055	Cell-substrate junction	138/1848	432/19869	2.02E−38	12
Macrophage	CC	GO:0005840	Ribosome	95/1848	232/19869	3.38E−36	12
Macrophage	CC	GO:0030139	Endocytic vesicle	109/1848	343/19869	5.72E−30	12
Macrophage	CC	GO:0031983	Vesicle lumen	103/1848	327/19869	5.83E−28	5
Macrophage	CC	GO:0034774	Secretory granule lumen	102/1848	322/19869	5.86E−28	12
Macrophage	CC	GO:0060205	Cytoplasmic vesicle lumen	102/1848	325/19869	1.24E−27	13
Macrophage	MF	GO:0003735	Structural constituent of ribosome	89/1830	176/18432	8.37E−40	11
Macrophage	MF	GO:0023023	MHC protein complex binding	24/1830	36/18432	1.50E−13	5
Macrophage	MF	GO:0023026	MHC class II protein complex binding	19/1830	27/18432	2.89E−11	11
Macrophage	MF	GO:0045296	Cadherin binding	75/1830	333/18432	2.02E−09	9
Macrophage	MF	GO:0140375	Immune receptor activity	44/1830	148/18432	2.84E−09	9
Macrophage	MF	GO:0009055	Electron transfer activity	39/1830	122/18432	2.92E−09	9
Macrophage	MF	GO:0004857	Enzyme inhibitor activity	78/1830	395/18432	3.39E−07	7
Macrophage	MF	GO:0042287	MHC protein binding	18/1830	42/18432	3.42E−06	9
Macrophage	MF	GO:0044389	Ubiquitin-like protein ligase binding	64/1830	318/18432	3.42E−06	4
Macrophage	MF	GO:0061134	Peptidase regulator activity	50/1830	232/18432	1.09E−05	6
Monocyte	BP	GO:0050863	Regulation of T cell activation	99/1124	376/18903	4.71E−34	99
Monocyte	BP	GO:0022407	Regulation of cell–cell adhesion	113/1124	490/18903	1.12E−33	113
Monocyte	BP	GO:0002443	Leukocyte mediated immunity	109/1124	463/18903	2.01E−33	109
Monocyte	BP	GO:0007159	Leukocyte cell–cell adhesion	102/1124	414/18903	4.79E−33	102
Monocyte	BP	GO:1903037	Regulation of leukocyte cell–cell adhesion	95/1124	377/18903	1.50E−31	95
Monocyte	BP	GO:0002253	Activation of immune response	95/1124	397/18903	1.12E−29	95
Monocyte	BP	GO:0050867	Positive regulation of cell activation	102/1124	467/18903	1.61E−28	102
Monocyte	BP	GO:0002696	Positive regulation of leukocyte activation	99/1124	450/18903	6.12E−28	99
Monocyte	BP	GO:0001819	Positive regulation of cytokine production	103/1124	486/18903	9.07E−28	103
Monocyte	BP	GO:0022409	Positive regulation of cell–cell adhesion	82/1124	322/18903	1.46E−27	82
Monocyte	CC	GO:0030666	Endocytic vesicle membrane	52/1155	194/19869	2.50E−18	52
Monocyte	CC	GO:0030139	Endocytic vesicle	70/1155	343/19869	3.25E−18	70
Monocyte	CC	GO:0070820	Tertiary granule	47/1155	164/19869	3.25E−18	47
Monocyte	CC	GO:0034774	Secretory granule lumen	65/1155	322/19869	9.41E−17	65
Monocyte	CC	GO:0060205	Cytoplasmic vesicle lumen	65/1155	325/19869	1.24E−16	65
Monocyte	CC	GO:0031983	Vesicle lumen	65/1155	327/19869	1.45E−16	65
Monocyte	CC	GO:0005925	Focal adhesion	74/1155	422/19869	8.33E−16	74
Monocyte	CC	GO:0030055	Cell-substrate junction	74/1155	432/19869	2.78E−15	74
Monocyte	CC	GO:0030667	Secretory granule membrane	61/1155	313/19869	3.06E−15	61
Monocyte	CC	GO:0101002	Ficolin-1-rich granule	45/1155	185/19869	6.77E−15	45
Monocyte	MF	GO:0023023	MHC protein complex binding	19/1143	36/18432	2.98E−11	19
Monocyte	MF	GO:0140375	Immune receptor activity	36/1143	148/18432	3.92E−10	36
Monocyte	MF	GO:0023026	MHC class II protein complex binding	15/1143	27/18432	1.95E−09	15
Monocyte	MF	GO:0003823	Antigen binding	34/1143	171/18432	2.89E−07	34
Monocyte	MF	GO:0042605	Peptide antigen binding	14/1143	37/18432	3.53E−06	14
Monocyte	MF	GO:0042287	MHC protein binding	14/1143	42/18432	1.74E−05	14
Monocyte	MF	GO:0005126	Cytokine receptor binding	41/1143	273/18432	1.74E−05	41
Monocyte	MF	GO:0042277	Peptide binding	46/1143	330/18432	2.37E−05	46
Monocyte	MF	GO:0004857	Enzyme inhibitor activity	52/1143	395/18432	2.37E−05	52
Monocyte	MF	GO:0042288	MHC class I protein binding	10/1143	22/18432	2.54E−05	10

### Cellular communication

**Fig. 5:**
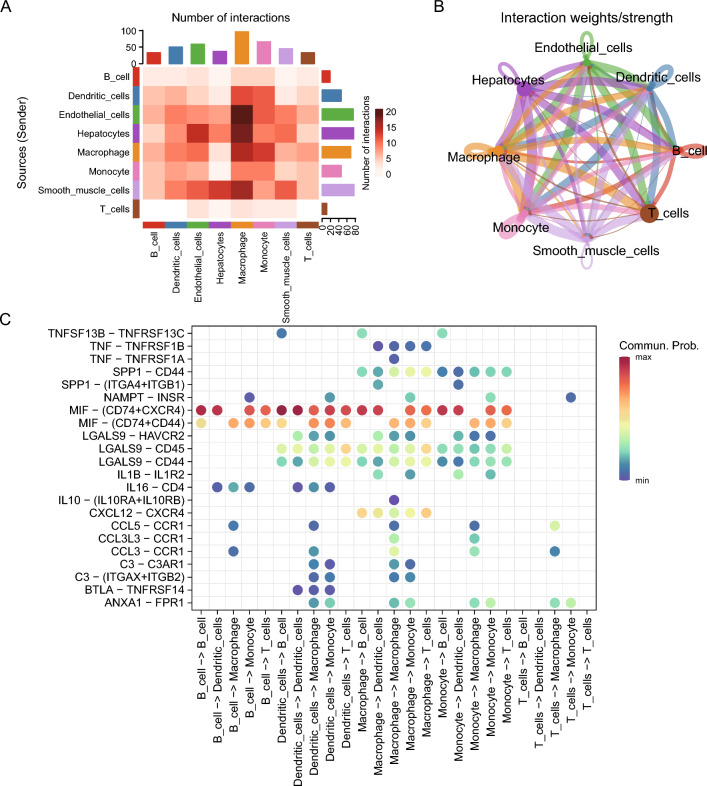
Single-cell dataset GSE149614 LIHC cell subpopulation communication analysis. **A** Heat map of the number of interactions between the 8 cell types, the darker shades of red indicated a higher number of interacting ligand-receptor pairs. **B** Network diagram of the intensity of intercellular interactions between 8 cell types, where nodes indicate various cell types, arrows indicate from the signal source cells to the receiving cells, and the line thickness indicates the intensity of the intercellular interaction. The thicker it is, the higher the intensity of the interaction. Different colors represent different cell types. **C** Ligand–receptor pairs of intercellular communication relationships between immune cell populations, where the horizontal coordinates indicate cell types where cell communication occurs, and the vertical coordinates indicate ligand-receptor pairs. The figure only displays communication relationship results that are statistically significant (P < 0.05). The color of the circle, from blue to red, denotes a gradual increase in the communication probability of cellular interactions

Cellular communication among 8 cell types was inferred and quantified by CellChat. In addition, the number (Fig. 5A) and intensity (Fig. 5B) of cellular communication were visualized by heat map and circle plot. Macrophages interacts with endothelial cells, hepatocytes, and smooth muscle cells in high numbers. In addition, hepatocytes and endothelial cells have high interaction intensity with macrophages and monocytes, respectively. In addition, all important receptor–ligand pairs (Fig. 4C) were counted when immune cells send/receive signals. MIF signaling pathway-related ligand-receptor pairs play a crucial role in this process (p < 0.01).

### Differential expression and correlation analyses of FRGs in the TCGA-LIHC dataset

**Fig. 6:**
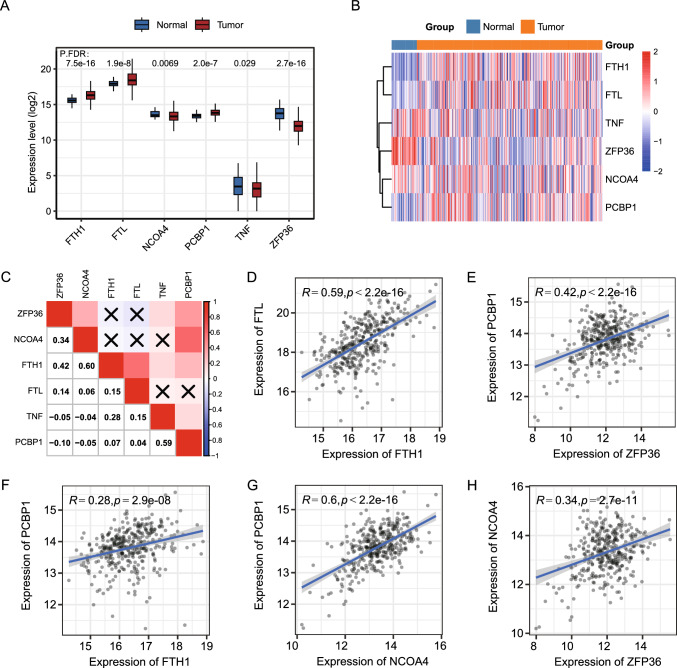
Expression of FRGs in TCGA-LIHC dataset and correlation analysis. **A** Box plot of the comparative expression levels of iron-related autophagy genes between LIHC tumors and paracancerous tissues. Group differences were analyzed by the Wilcoxon test, FDR-corrected p-values were annotated on the graph; **B** Differential expression of FRGs between different samples, shown as a heat map; orange represents tumor group, blue represents paracancer control group. The color of gene expression levels from light to dark indicates elevated expression levels, with the negative association in blue and positive in red. **C** Correlation matrix of differentially expressed FRG expression levels. Red denotes a positive correlation, and blue denotes a negative correlation. Darker colors indicate enhanced correlations, and non-significant ones are shown by black X. **D**–**H** Correlation analysis of expression levels of ferritinophagy with significant results, where correlation coefficients above 0.2 are indicated by dotted line plots, and correlation coefficients R and P values are labeled on the plots, respectively

The varied expression levels of DEFRGs between the tumor and control groups in the TCGA-LIHC liver cancer dataset were compared, where ZFP36, NCOA4, FTH1, FTL, TNF, and PCBP1 were matched with the TCGA transcriptome data. The expression of all six FRGs was significantly different (p < 0.05), with FTH1, FTL, and PCBP1 being overexpressed in the tumor group and ZFP36, NCOA4, and TNF expressed less in the tumor group (Fig. 6A). The expression levels of FRGs in different samples were presented as a heat map (Fig. 6B). The correlation matrix of FRGs expression levels was shown in Fig. 6C, where PCBP1 had a considerable positive association with ZFP36, NCOA4, and FTH1, respectively, and ZFP36 and NCOA4 and FTH1 and FTL had a considerable positive association, respectively. The correlation results with correlation coefficients greater than 0.2 were presented as scatter plots, in which FTL was positively linked with FTH1 (R = 0.59, P < 0.001), PCBP1 was positively associated with ZFP36 (R = 0.42, P < 0.001), PCBP1 was positively associated with FTH1 (R = 0.28, P < 0.001), PCBP1 was positively associated with NCOA4 (R = 0.60, P < 0.001), and NCOA4 was positively associated with ZFP36 (R = 0.34, P < 0.001).

### Assessment of the immune microenvironment and correlation analysis of FRGs

**Fig. 7:**
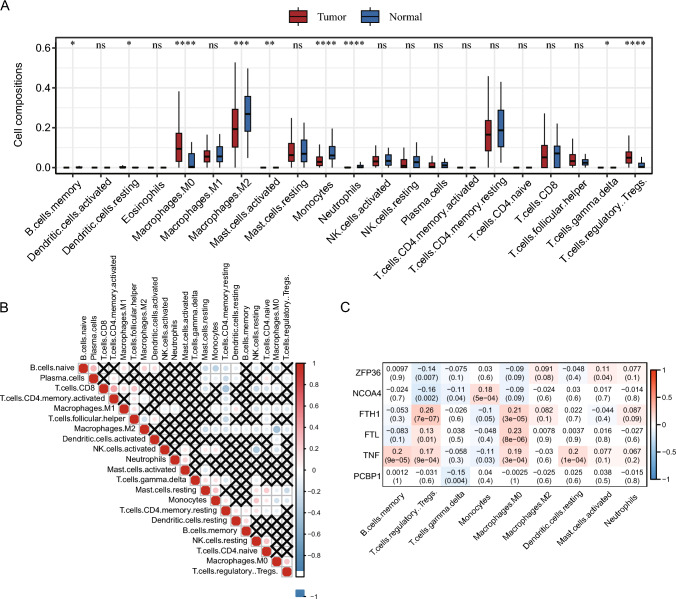
Immune cell infiltration analysis of TCGA-LIHC dataset. **A** Comparison of different levels of immune cell infiltration in the LIHC case/control group. Differences between groups were analyzed by the Wilcoxon test, and the statistical significance of differences is indicated by the "*" sign, where "*" indicates P < 0.05, "**" indicates P < 0.01, "***" indicates P < 0.001, "****" indicates P < 0.0001; **B** correlation matrix between immune cells, where red denotes positive association, blue denotes a negative association and darker color indicates increased association. Non-statistical significance is indicated by black X's; **C** Correlation matrix between immune cells and FRGs, where red denotes a positive association, blue indicates a negative correlation, and darker color indicates enhanced correlation, and correlation coefficients and p-values are marked in squares

The infiltration of different immune cells in the TCGA-LIHC dataset was analyzed using CIBERSORTx. The infiltration of 22 immune cells among different subgroups is illustrated in Fig. 7A. M2 macrophages, neutrophils, monocytes, memory B cells, and gamma delta T cells were less infiltrated in the LIHC case set, while M0 macrophages, Tregs, resting dendritic cells, and activated mast cells were more infiltrated in the LIHC case group. Correlations between the degree of infiltration between different immune cells were analyzed, and the correlation matrix is shown in Fig. 7B. where cells with correlation coefficients greater than 0.3 or less than − 0.3 were selected. CD8 T cells and activated memory CD4 T cells were positively correlated (coefficient = 0.40, P < 0.001), and naïve B cells and plasma cells were negatively associated (coefficient = 0.33, P < 0.001), M2 macrophages and M0 macrophages were negatively associated (coefficient = − 0.45, P < 0.001), resting memory CD4 T cells and CD8 T cells were negatively associated (coefficient = − 0.44, P < 0.001), naïve B cells and monocytes were negatively associated (coefficient = − 0.33, P < 0.001), resting and activated NK cells were negatively associated (coefficient = − 0.38, P < 0.001). Finally, the association of DEFRGs with different immune cells was assessed separately (Fig. 7C). Most FRGs were observed to have a significant positive correlation with M0 macrophages and Tregs (P < 0.001).

### WGCNA

**Fig. 8:**
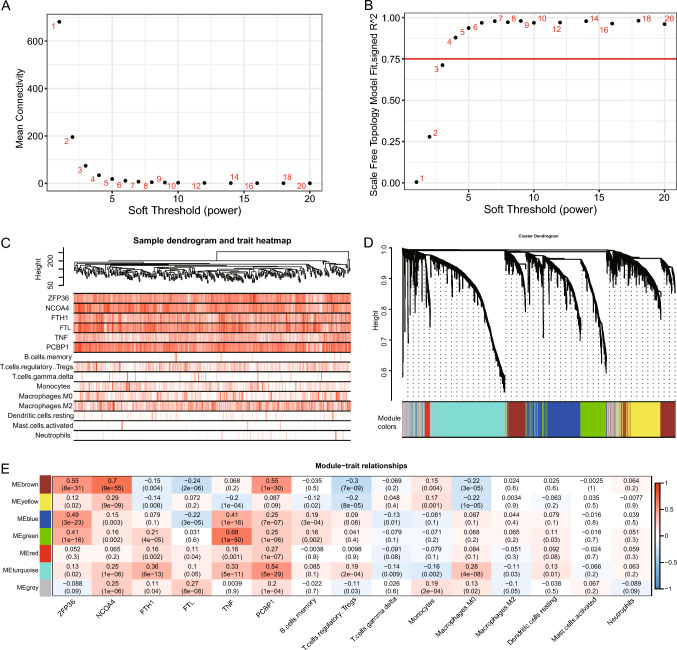
WGCNA of TCGA-LIHC dataset. **A**, **B** The power parameter screening process of WGCNA, where the values of clustering tree Connectivity **A** and model fitting R-square **B** with increasing power and slowing down the rate of change is the best power value, suggesting that 4 is the best power value; **C** TCGA-LIHC dataset **C** tree diagram of cluster analysis of samples and corresponding FRGs-related gene expression and immune cell infiltration; **D** gene cluster analysis of WGCNA illustrated as a tree diagram, where the modules of gene classification are demonstrated by distinct colors; **E** correlation matrix between the module scores retrieved by WGCNA and the obtained FRGs-related gene expression and the level of differential immune cell infiltration, with red indicating positive correlation, green indicates negative correlation. Darker colors indicate an enhanced correlation, and correlation coefficients and p-values are shown in the cells within the matrix. *WGCNA* Weighted Gene Co-Expression Network Analysis, *FRGs* ferritinophagy-related genes

In total, 3882 DEGs, including 1588 overexpressed genes and 2294 genes with low expression, were screened from the TCGA-LIHC gene expression matrix utilizing the limma package of R language. The screened DEGs were accepted by WGCNA (Fig. 8). Seven modules were calculated, which had some correlation with FRGs and immune cell infiltration, respectively. MEbrown had a considerably positive link with ZFP36, NCOA4, and PCBP1 (P < 0.001), MEgreen had a considerably positive link with TNF (P < 0.001), MEturquoise had a considerably positive link with PCBP1 (P < 0.001), and MEbrown and MEyellow had a considerably positive association with Tregs and macrophages, respectively. M2 had a considerable negative correlation (P < 0.001), and MEturquoise had a considerable positive correlation with M0 macrophages (P < 0.001).

### Correlation between DEFRGs and patient prognosis

**Fig. 9:**
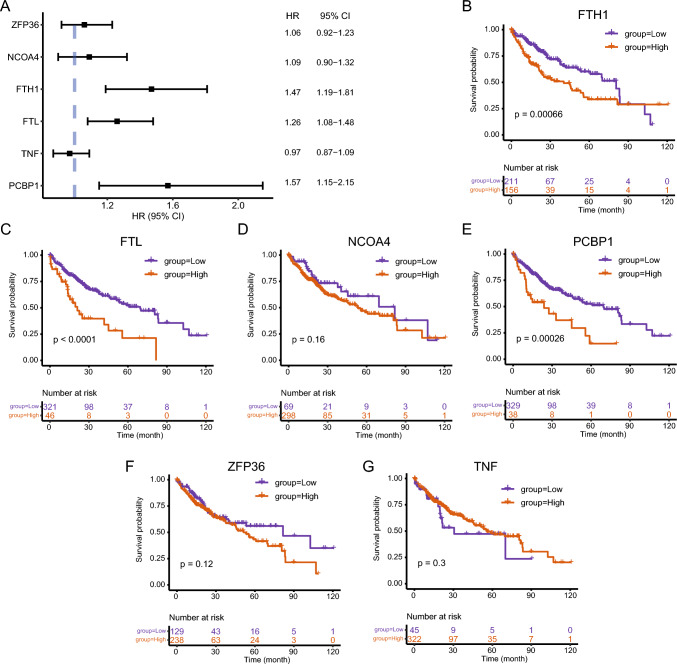
Survival analysis of risk groups for the TCGA-LIHC dataset. **A** Forest plot of the outcomes of COX regression analysis for the six DEFRGs. **B**–**G** Survival curves (K–M method) for FTH1 (**B**), FTL (**C**), NCOA4 (**D**), PCBP1 (**E**), ZFP36 (**F**), and TNF (**G**) in high- and low-expression groups. Cutoff values were determined by the maxstat package, where orange reaches the high-risk group and purple color denotes the low-risk group. FRGs. FRGs, *K–M* Kaplan–Meier

The association between DEFRGs in the TCGA-LIHC dataset and the patient prognosis was analyzed using univariate Cox regression. Patients with higher expression of FTH1 (HR = 1.47, 95% CI 1.19–1.81), FTL ((HR = 1.26, 95% CI 1.08–1.48), and PCBP1 (HR = 1.57, 95% CI 1.15–2.15) had a poorer prognosis (Fig. 9A). Individuals were categorized into high- and low-expression groups as per the gene expression using the maxstat package, and the results were in line with the outcomes of COX regression analysis with continuous variables. Individuals with high expression of FTH1, FTL, and PCBP1 had a considerably poorer prognosis than those with low expression of these genes (P < 0.001).

### Prognostic marker screening and risk score construction

**Fig. 10:**
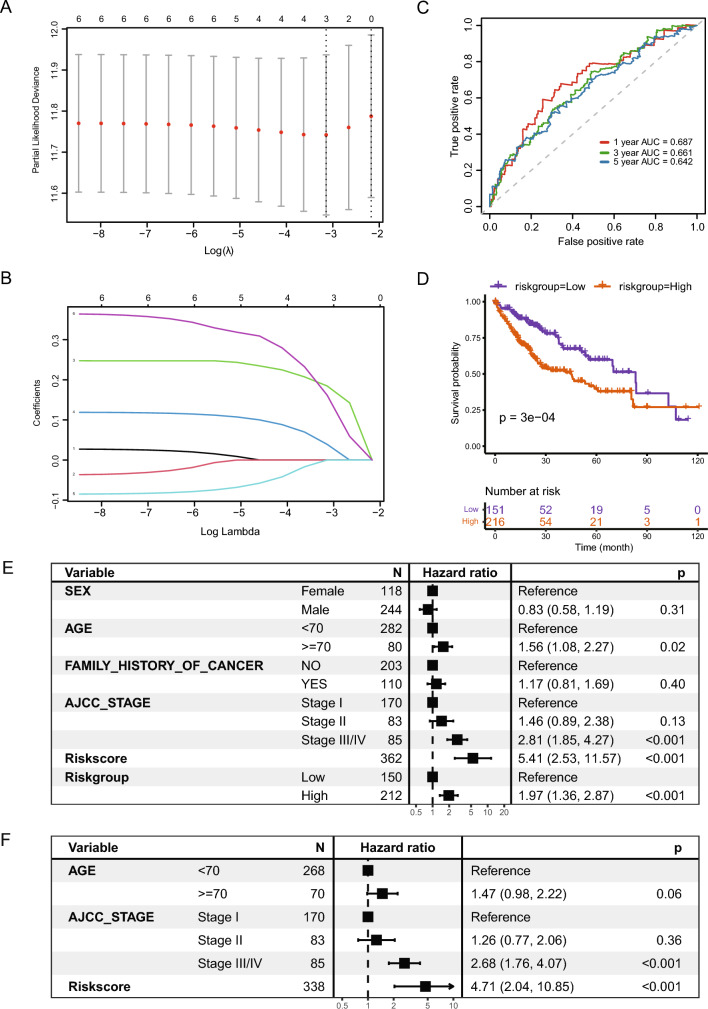
LASSO regression screening for prognosis-related FRGs. **A**, **B** Screening of prognostic markers using LASSO logistic regression models; partial likelihood deviation with tenfold cross-validation used to calculate the optimal λ; **C** ROC curves and AUC values for risk score prediction of 1-, 3- and 5-year survival of patients; **D** Survival curves for high and low expression groups according to risk scores (K–M method); **E** Other COX regression analysis of the impact of clinical features on patient prognosis, presented as a forest plot; **F** Multivariate COX regression analysis of significant clinical features in C, presented as a forest plot. *LASSO*, absolute shrinkage and selection operator, *FRGs* ferritinophagy-related genes, *ROC* receiver operating characteristic *AUC* area under the curve, *K–M* Kaplan–Meier

LASSO regression analysis was utilized to screen three FRGs as prognostic markers, including FTH1, FTL, and PCBP1 (Fig. 10A, B).

The coefficients of the candidate prognostic markers were found based on the results of the analysis of the LASSO regression model. Subsequently, the risk score RS was measured by means of the following equation:

$$\mathrm{Risk score}=0.1846*\mathrm{ FTH}1+ 0.0391*\mathrm{ FTL}+0.1618*\mathrm{ PCBP}1$$.

The ROC curves for risk score prediction of 1-, 3- and 5-year survival of diseased individuals are shown in Fig. 10C, with the best predictive power for 1-year survival (AUC = 0.687). The best cutoff for the predictive ability of the risk score for survival time in individuals with LIHC was determined using the maxstat package was 5.9135. Individuals with LIHC were categorized into high- and low-risk groups as per their cutoff values. In addition, individuals with no survival data were eliminated. Individuals with high risk scores had significantly shorter prognostic survival duration than those with low risk scores (Fig. 10D).

### Prognostic model construction

**Fig. 11:**
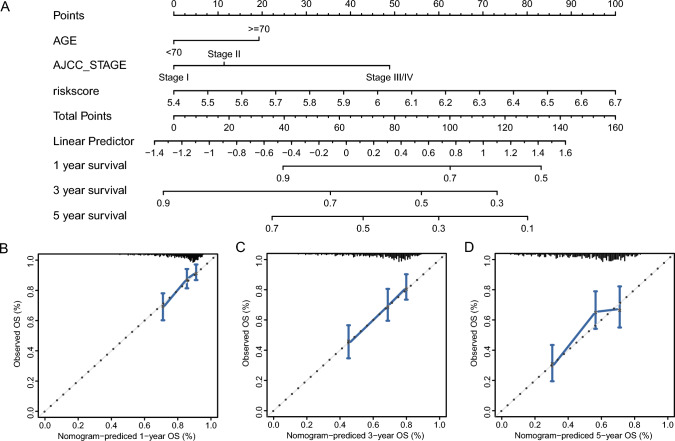
Prognostic risk model for LIHC. **A** Nomogram of the multivariate COX regression model for risk score prediction of survival in individuals with LIHC from the TCGA-LIHC dataset. **B**–**D** Calibration curves for 1-(**B**), 3-(**C**), and 5-(**D**) year survival prediction

The outcomes of univariate COX regression demonstrated that both age and tumor stage affected patient survival except for risk score/group (Fig. 10E). A multivariate prognostic prediction model was constructed using the Cox regression model (Fig. 10F). Nomogram (Fig. 11A) and calibration curves (Fig. 11B–D) were drawn with the rms package for predicting 1-, 3- and 5-year survival in individuals with LIHC. Tumor stage, age (c-index = 0.633), and risk score (c-index = 0.635) were used to construct the nomogram, which demonstrated their value as predictors. With a c-index of 0.678, the survival prediction model constructed by integrating age, stage, and risk scores demonstrated superior prognosis predictive performance.

### Immunophenotypes of risk groups

**Fig. 12:**
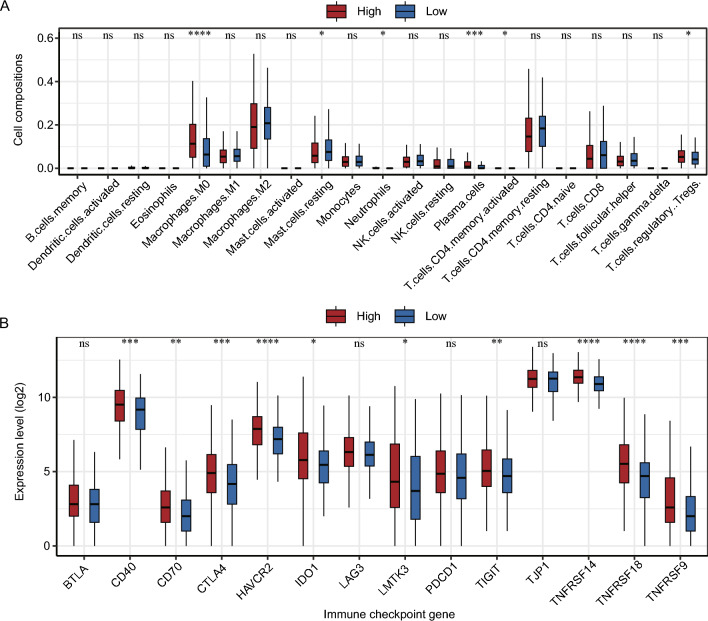
Immunophenotypes of risk groups. **A** Infiltration of immune cells in the risk subgroups. **B** Expression of common immune checkpoint genes in the risk subgroups. Differences between groups were analyzed by the Wilcoxon test. Statistically significant differences are indicated by "*" signs, where P < 0.05 is indicated by "*", P < 0.01 by "**", and P < 0.001 by "***", P < 0.0001 by "****", insignificant by ns

A comparison of immune cell abundance between the risk subgroups in the TCGA-LIHC dataset is shown in Fig. 12A. M0 macrophages, plasma cells, and Tregs had higher abundance in the high-risk group (P < 0.05). Resting mast cells, neutrophils, and activated memory CD4 T cells had higher abundance in the low-risk group (P < 0.05).

In both the risk groups, the expression of 14 common immune check loci (BTLA, CD40, CD70, CTLA4, HAVCR2, IDO1, LAG3, LMTK3, PDCD1, TIGIT, TJP1, TNFRSF14, TNFRSF18, and TNFRSF9) was observed. CD40, CD70, CTLA4, HAVCR2, IDO1, LMTK3, TIGIT, TNFRSF14, TNFRSF18, and TNFRSF9 had substantially different levels of expression between the two risk groups (P < 0.05) (Fig. 12B).

### Drug sensitivity prediction

**Fig. 13:**
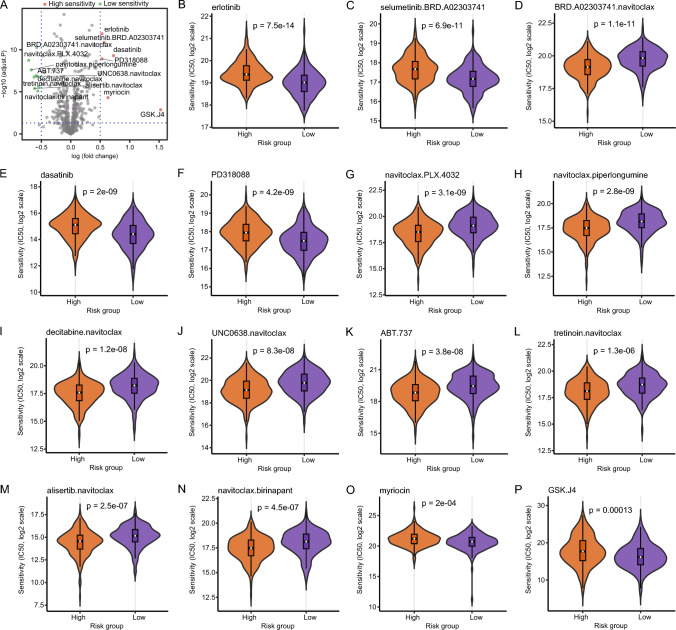
Drug sensitivity variation in high- and low-risk groups. **A** Drugs with differences are shown in volcano plots, with red and green indicating higher and lower drug sensitivity, respectively, in the group with high risk. In addition, adj.p value < 0.05 and |log2FC|> 0.5 suggest substantial variations in drug sensitivity among the risk groups; **B**–**P** Box plots showing variation in drug sensitivity between the risk groups for erlotinib (**B**), selumetinib. Selumetinib.BRD.A02303741 (**C**), BRD.A02303741.navitoclax (**D**), dasatinib (**E**), PD318088 (**F**), navitoclax.PLX.4032 (**G**), navitoclax.piperlongumine (**H**), decitabine.navitoclax (**I**), UNC0638.navitoclax (**J**), ABT.737 (**K**), tretinoin.navitoclax (**L**), alisertib.navitoclax (**M**), navitoclax. birinapant (**N**), myriocin (**O**), and GSK.J4 (**P**), where orange and purple represent the groups with high and low risk, respectively

According to drug sensitivity analysis, a significant difference was observed in drug sensitivity to erlotinib (Fig. 13B) and selumetinib.BRD.A02303741 (Fig. 13C), BRD.A02303741.navitoclax (Fig. 13D), dasatinib (Fig. 13E), PD318088 (Fig. 13F), navitoclax.PLX.403 (Fig. 13G), navitoclax.piperlongumine (Fig. 13H), decitabine.navitoclax (Fig. 13I), UNC0638.navitoclax (Fig. 13J), ABT.737 (Fig. 13K), tretinoin.navitoclax (Fig. 13L), alisertib.navitoclax (Fig. 13M), navitoclax.birinapant (Fig. 13N), myriocin (Fig. 13O), and GSK.J4 (Fig. 13P) between both risk groups (Fig. 13A, adj.*p value* < 0.05 and |log_2_FC|> 0.5). Individuals in the group with high risk were more sensitive to GSK.J4, dasatinib, myriocin, and selumetinib. BRD.A02303741, erlotinib, and PD318088 individuals in the group with low risk. However, individuals in the group with high risk were less sensitive to tretinoin.navitoclax, navitoclax.birinapant, UNC0638.navitoclax, BRD. A02303741. Navitoclark, decitabine.navitoclax, alisertib.navitoclax, ABT.737, navitoclax.piperlongumine, and navitoclax.PLX.4032 than those in the low-risk group.

### Somatic mutation analysis of risk groups

**Fig. 14:**
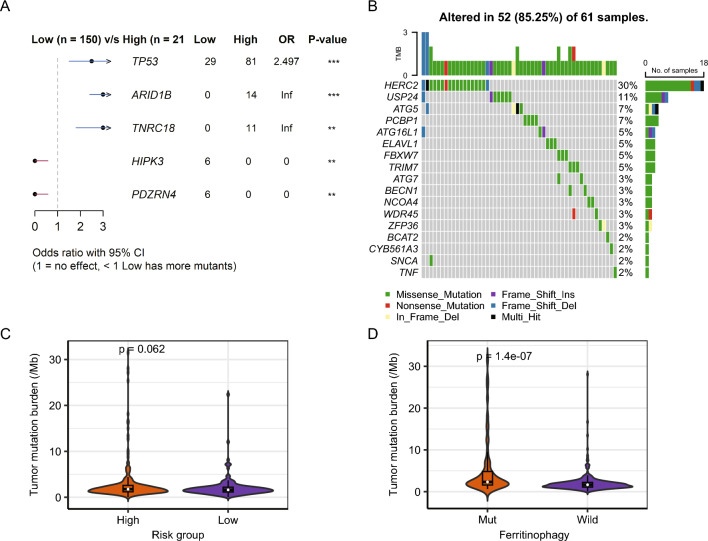
Somatic mutation analysis of the TCGA-LIHC dataset. **A** Fisher's exact analysis of the differences in somatic mutations between both risk groups. **B** Waterfall plot of mutations in FRGs. **C** Violin plot of the number of Tmbs in the risk groups, where the horizontal coordinate denotes the two risk groups and the vertical coordinate denotes the TMB. The variations between groups were analyzed by the Wilcoxon test. **D** Violin plot of the number of Tmbs in the FRG mutation/non-mutation groups, where the horizontal coordinate denotes the risk group and the vertical coordinate denotes the TMB. The Wilcoxon signed-rank test was utilized to observe group variations

Fisher's exact test for somatic mutations was performed to detect differentially mutated genes in tumor samples between groups with high- and low-risk in the TCGA-LIHC dataset (2 cases with no mutation data and 366 cases with analyzed data). The genes with the most significant differences were TP53, ARID1B, TNRC18, HIPK3, and PDZRN4 (P < 0.01, Fig. 14A). Subsequently, mutations in FRGs were counted, with the highest mutation frequency being HERC2 (23/366, 6.28%), followed by USP24 (8/366, 2.18%), ATG5 (6/366, 1.63%), and PCBP1 (4/366, 1.09%) (Fig. 14B). Moreover, the number of Tmbs was counted between the risk groups and between FRG mutation/non-mutation groups. The number of Tmbs did not differ considerably between the risk groups (Fig. 14C), whereas the number of Tmbs was substantially larger in the FRG mutation group than that in the non-mutation group (Fig. 14D).

### Somatic mutation features of risk groups

**Fig. 15:**
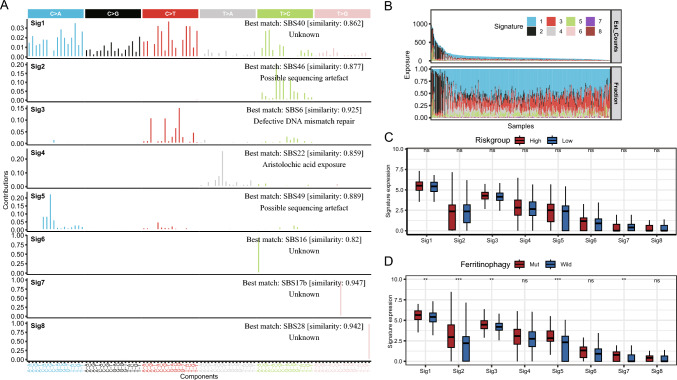
Somatic mutation feature analysis of the TCGA-LIHC dataset. **A** Mutation patterns of somatic mutation features of LIHC samples and similar mutation features of the COSMIC database. **B** Composition of the mutation features of the samples. **C** Expression of each mutation feature in both risk groups. **D** Expression of each mutation feature in FRG mutation and non-mutation subgroups. Wilcoxon test was utilized to examine variations between groups. Statistically significant differences are indicated by "*" signs, where *P < 0.05, **P < 0.01, ***P < 0.001, ****P < 0.0001, and ns represents insignificant. In addition, red and blue denote the high– and low-risk groups, respectively. COSMIC, the Catalogue of Somatic Mutations in Cancer

A total of eight mutation features were extracted from the TCGA-LIHCLIHC somatic mutation data (Fig. 15A). Among them, Sig1 is similar to SBS40 in the COSMIC database (unknown pathogen), Sig2 is similar to SBS46 (sequencing artifact), Sig3 is similar to SBS6 (MMR disorder), Sig4 is similar to SBS22 (aristolochic acid), Sig5 is similar to SBS49 (sequencing artifact), Sig6 is similar to SBS16 (unknown pathogen), Sig7 is similar to SBS17b (unknown pathogen), and Sig8 is similar to SBS28 (unknown pathogen). Sig1 and Sig2 had a higher prevalence of somatic mutations in most samples (Fig. 15B). The expression of mutant characteristics was then compared between the risk groups as well as between the FRG mutation/non-mutation groups. No difference in the expression of the mutation features was observed between the high- and low-risk groups (P > 0.05) (Fig. 15C). However, the FRG mutation group exhibited considerably greater levels of Sig1, Sig2, Sig3, Sig5, and Sig7 expression than the non-mutation group (P < 0.01) (Fig. 15D).

### FTH1,FTL is highly expressed in hepatocellular carcinoma cells and hepatocellular carcinoma tissues.

**Fig. 16:**
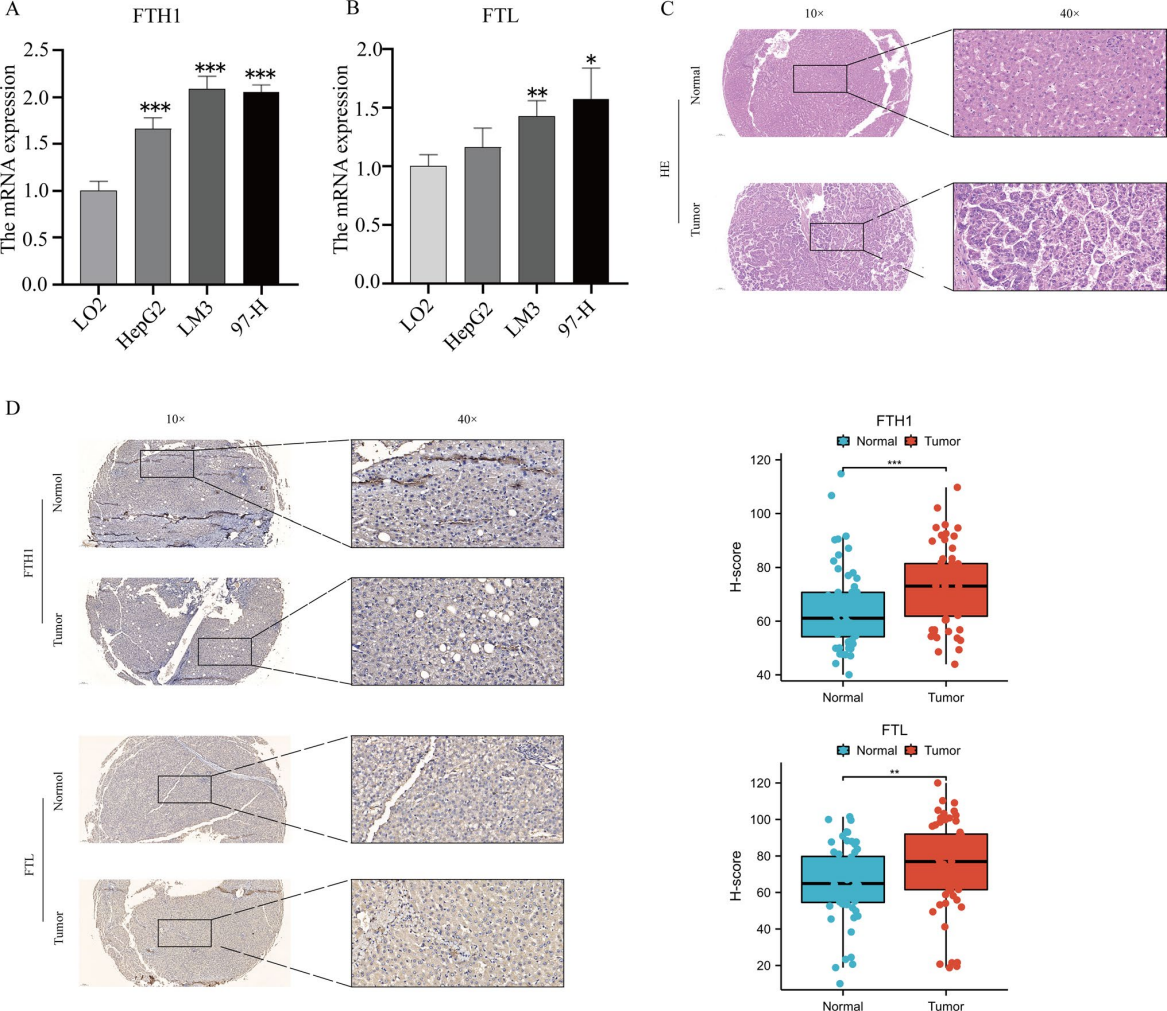
**A** expression of FTH1 mRNA in different liver cancer cells and normal liver cells; **B** expression of FTL mRNA in different liver cancer cells and normal liver cells; **C** the HE staining results of the patient tissue samples. **D** expression of FTH1, FTL in tumor tissue; *P < 0.05, **P < 0.01, ***P < 0.001

We further validated the FTH1, FTL genes that were significantly associated with poor prognosis. We conducted PCR experiments using a normal liver cell line (LO2) and three liver cancer cell lines (HepG2, LM3, and 97-H). As shown in Fig. 16A, B, FTH1, FTL were significantly overexpressed in the three liver cancer cell lines compared with normal liver cells.

We first performed HE staining on the patient tissue specimens we collected to distinguish between cancer tissue and adjacent normal tissue. (Fig. 16C). We further detected 60 pairs of liver cancer tissues by immunohistochemistry, and found that the expression of FTH1, FTL in tumor tissues was significantly higher than that in para-tumor tissues. This is consistent with previous bioinformatics results (Fig. 16D).

## Discussion

This study is a pioneering effort integrating TCGA and single-cell databases to investigate the expression, prognosis, mutation, and immune infiltration associated with FRGs in LIHC. Additionally, prognostic indicators based on DEFRGs were screened using LASSO regression analysis. Finally, a survival prediction model was constructed by integrating age, stage, and risk scores. The nomogram and calibration curves revealed that the model had excellent prognosis predictive performance.

The rapid advancement of single-cell technology has deepened the understanding of tumorigenesis [36, 37]. The extracted cells were classified into eight different cell types. Furthermore, the DEGs among immune cells intersected with the extracted FRGs, yielding 7 DEGs. A total of 6 genes were found to be differentially expressed according to a TCGA database search. Additionally, in line with earlier studies, FTH1, FTL, and PCBP1 could be used as prognostic markers [38–40]. Ferroptosis is a lysosome-dependent autophagic cell death process [41, 42]. Ferroptosis and autophagy are mutually reinforcing, with ferritinophagy being the biological process at the intersection of the two [43]. Ferroptosis and autophagy are actively involved in cancer progression. ferritinophagy is also inextricably linked to the progression of cancer [44, 45]. A series of prognostic models based on the relationship between ferroptosis and LIHC could help to accurately monitor the progression of LIHC. In addition, ferroptosis-related gene models could predict the prognosis and the choice of treatment for LIHC patients. Moreover, ferroptosis-related prognostic models constructed based on some methylation profiles of LIHC could predict the associated risk more accurately [46–48]. In the present study, a model based on ferritinophagy-related prognostic markers was constructed. Individuals with high-risk scores exhibited considerably poorer survival rates compared to individuals with low-risk scores. Furthermore, a multivariate prognostic prediction model was constructed using a Cox regression model, which showed excellent prognostic predictive power.

TME consists of immune cells, non-immune stromal cells (including endothelial cells, fibroblasts, etc.), and extracellular matrix proteins, which impact the tumor process [49–51]. By secreting various cytokines, chemokines, and other signaling molecules that interact with cancer cells, different cells are vital in the control of the tumor immune response. Similarly, TME is crucial in the immune response of LIHC [52, 53]. In this study, multiple FRGs were found in the DEGs among immune cells. Expression scoring of DEGs indicated that monocytes and macrophages had the highest FRGs scores. In addition, various immune cell infiltration in the TCGA-LIHC dataset was evaluated. As expected, considerable variations in immune infiltration were observed in LIHC. The association of DEFRGs with different immune cells was further evaluated. Most FRGs showed significant positive correlations with M0 macrophages and Tregs. According to the models generated by DEFRGs, there were similar variations between high- and low-risk groups in immune cell abundance and expression of multiple immune checkpoints. Cellular communication analysis showed a higher number of interactions between macrophages and endothelial cells, hepatocytes, and smooth muscle cells, whereas hepatocytes and endothelial cells both interacted with macrophages and monocytes, respectively, with a high interaction intensity. Therefore, FRGs and the level of immune infiltration of LIHC were strongly associated. Monocytes and macrophages with high expression of FRGs are actively involved in the immune response of LIHC. Monocytes and macrophages are excellent potential therapeutic targets for LIHC [54, 55]. Since LIHC mainly progresses to fibrosis or cirrhosis, it has relatively low responsiveness to immune checkpoint blockade (ICB) therapy. In previous research, intrinsic enhancer reprogramming targeting monocytes improved the immunotherapeutic efficacy of LIHC [56]. Macrophages could be classified as M1 or M2 depending on their phenotype [57]. Macrophage polarization is influenced by the tumor stage and presents different polarization states depending on the tumor or intra-tumor region [58]. In addition, LIHC progression is considered associated with a skewed macrophage phenotype from M1 to M2 [59].

The instability of genetic material accelerates the acquisition of genetic diversity and is a hallmark feature that promotes cancer onset and progression [60]. Over 10,000 genes were found to have significant mutations in HCC, with 26 of them showing the highest mutation frequency, including TP53, CTNNB1, and AXIN1 [61]. This was validated in the current study. The number of Tmbs was counted for the low- and high-risk groups as well as the FRG mutation and non-mutation groups. No substantial difference was found in TMB between both risk groups. However, it was remarkably increased in the FRG mutation group than in the non-mutation group. High-mutation frequency genes, such as FRGs like HERC2 and USP24, have been linked to the onset and progression of various malignancies [62–65]. However, the relevance of these genes to LIHC needs to be explored in further studies.

The current study has certain limitations. First, public databases were used to collect the data for this investigation. Therefore, further validation using different external data sets is needed. Second, in order to further validate the findings of this study, in vitro and in vivo investigations are necessary. Finally, FRGs were defined by searching on GeneCards, which may introduce some bias. In summary, a LIHC-related prognostic model based on FRGs was constructed, which offers fresh insights into LIHC prevention and treatment.

## Conclusion

We investigated the relationship between ferritinophagy and the occurrence and development of LIHC. Through screening of differentially expressed FRGs that were significantly correlated with patient prognosis, we further constructed a relevant risk model. Further analysis revealed significant differences in terms of immune infiltration, immune checkpoints, drug sensitivity, TMB, etc., between the high-risk and low-risk model groups. Our in vitro PCR, IHC experiments also validated our research. In summary, our study provides a new research idea for the prevention and treatment of LIHC.

## Data Availability

The data that support the findings of this study are available on request from the corresponding author.

## Abbreviations

AUC: Area under the receiver operating characteristic curve
BH: Benjamini–Hochberg
BPs: Biological processes
CCs: Cell components
CGP: Cancer Genome Project
COSMIC: Catalogue of Somatic Mutations in Cancer
DEFRGs: Differentially expressed ferritinophagy-related genes
FDR: False discovery rate
FRGs: Ferritinophagy-related genes
GEO: Gene Expression Omnibus
GO: Gene ontology
IC50: Half-maximum inhibitory concentration
KEGG: Kyoto Encyclopedia of Genes and Genomes
LASSO: Least absolute shrinkage and selection operator
LIHC: Liver hepatocellular carcinoma
MFs: Molecular functions
PC: Principal component
PCA: Principal Component Analysis
qRT-PCR: Quantitative reverse transcription polymerase chain reaction
ROC: Receiver operating characteristic
ROS: Reactive oxygen species
scRNA-seq: Single-cell RNA sequencing
TCGA: The Cancer Genome Atlas
TMB: Tumor mutation burden
TME: Tumor microenvironment
tSNE: T-Distributed Stochastic Neighbor Embedding
WGCNA: Weighted Gene Co-Expression Network Analysis
